# Evaluating Hospital Efficiency and Productivity: A Bootstrap-Corrected DEA and Malmquist Analysis

**DOI:** 10.3390/healthcare14183084

**Published:** 2026-09-19

**Authors:** Charalampos Poriazis, Maria Katharaki, Nikos Giannakis, Paraskevi Boufounou, Marios Tsakas

**Affiliations:** 1 Department of Agricultural Development, Agri-Food and Natural Resources Management, National and Kapodistrian University of Athens, 34400 Athens, Greece; chporiazis@agro.uoa.gr (C.P.); nvgiannakis@agro.uoa.gr (N.G.); tsakasm@agro.uoa.gr (M.T.); 2 Department of Nursing, National and Kapodistrian University of Athens, 11527 Athens, Greece; 3 Department of Economics, National and Kapodistrian University of Athens, 10559 Athens, Greece; pboufounou@econ.uoa.gr

**Keywords:** Data Envelopment Analysis, bootstrap, Simar–Wilson bootstrap truncated regression, Malmquist Productivity Index, hospital efficiency, public hospitals, Greece

## Abstract

**Highlights:**

**What are the main findings?**

- Bootstrap-corrected DEA revealed substantial variation in volume-based technical efficiency, while scale-related effects contributed to inefficiency in some hospitals.
- Population ageing, population density, and unemployment were negatively associated with efficiency, while productivity declined overall with substantial year-to-year variation.

**What are the implications of the main findings?**

- DEA can support structured regional performance review, but efficiency scores should not be interpreted as stand-alone hospital rankings or uniform resource-reduction targets.
- Performance assessment should combine efficiency with case mix, quality of care, accessibility, population need, and local service responsibilities.

**Abstract:**

**Background/Objectives:** Efficient resource allocation is essential for the sustainability of public hospitals operating under financial and capacity constraints. This study evaluated the volume-based technical efficiency and productivity of public hospitals within a Greek Health Region and explored associations between efficiency and selected contextual characteristics. **Methods:** Of 15 public hospitals in the study setting, two were excluded on production-comparability grounds, leaving 13 hospitals analyzed over 2015–2021 using input-oriented Data Envelopment Analysis under constant and variable returns to scale. Bootstrap bias correction was applied to CRS efficiency estimates, while scale efficiency was derived from CRS and VRS scores. Exploratory associations between 2021 bias-corrected efficiency and population ageing, population density, and unemployment were examined using Simar–Wilson double-bootstrap truncated regression. Productivity change was assessed using the Malmquist Productivity Index. **Results:** Mean bias-corrected CRS efficiency ranged from 0.7486 to 0.8556, and no hospital achieved a bias-corrected score of 1. Mean scale efficiency ranged from 0.8981 to 0.9416. Population ageing, population density, and unemployment were negatively associated with efficiency in the primary second-stage specification, with coefficient directions remaining broadly consistent across sensitivity models. Malmquist analysis indicated an overall decline in productivity, driven predominantly by shifts in the estimated production frontier, with substantial year-to-year variation. **Conclusions:** Hospital efficiency and productivity varied substantially across hospitals and over time. DEA can support regional performance review, but results should be interpreted alongside case mix, quality, accessibility, population need, and local service responsibilities rather than as stand-alone performance rankings.

## 1. Introduction

Health systems worldwide face increasing pressure to provide accessible and effective services within limited financial, human, and infrastructural resources. These pressures are particularly severe in countries experiencing prolonged fiscal constraints, where public hospitals must maintain service provision while operating under restricted public budgets [1,2]. Efficient resource allocation is therefore central to the sustainability of hospital operations and to the ability of health systems to maintain service provision over time [3,4,5].

The Greek healthcare system combines a tax-funded National Health System, compulsory social insurance, and private healthcare provision [6]. Public hospitals fall under the central oversight of the Ministry of Health and are supervised locally by Regional Health Authorities. They rely on two principal funding sources: direct allocations from the state budget and reimbursements from the National Organization for Healthcare Services Provision. Although the National Health System was designed to ensure universal and equitable access, Greek public hospitals continue to face persistent financial, staffing, and infrastructure constraints. These constraints are compounded by geographical inequalities in the distribution of personnel, beds, infrastructure, and healthcare demand [7,8]. In 2022, healthcare expenditure in Greece represented approximately 8.6% of gross domestic product and amounted to around EUR 2750 per capita, compared with an OECD average of approximately EUR 4600 [9,10]. Previous research has also identified limitations in medical equipment and hospital capacity [11,12]. More recently, declining patient visits, a reduction in medical personnel, and substantial regional inequalities in service utilization, staffing, and infrastructure were reported for the period 2018–2023 [13]. These pressures make it increasingly important to understand how effectively hospitals use available resources to deliver healthcare services.

Partial indicators, such as patients per bed or expenditure per patient, provide useful information but assess only individual relationships between inputs and outputs. Hospitals use several resources simultaneously, including beds, personnel, and expenditure, to produce multiple services. A hospital may therefore perform well according to one measure and poorly according to another, making overall comparisons difficult [14,15].

Frontier-based methods provide a more comprehensive approach to hospital performance assessment. Parametric methods require assumptions regarding the functional relationship between resources and outputs, and misspecification may affect the resulting estimates [16]. Data Envelopment Analysis (DEA), by contrast, is a non-parametric method that evaluates the relative efficiency of comparable decision-making units using multiple inputs and outputs without imposing a predetermined production function [17]. It has been widely used to evaluate the efficiency of hospitals and other healthcare organizations [7,18,19,20,21,22,23].

Nevertheless, DEA estimates may be affected by sampling variation and upward bias because the efficiency frontier is constructed from the observed sample. Bootstrap procedures can improve statistical reliability by producing bias-corrected estimates and confidence intervals. In addition, first-stage DEA scores do not explain how socioeconomic or environmental conditions are associated with efficiency. Second-stage regression models can therefore be used to examine factors external to hospital production. Static efficiency estimates do not indicate how hospital productivity changes over time. The Malmquist Productivity Index addresses this by decomposing productivity change into efficiency change and technological change.

The choice of DEA specification also affects interpretation [24]. Under constant returns to scale (CRS), efficiency scores capture overall technical efficiency [17] and may reflect both operational performance and the scale at which a hospital operates. Variable returns to scale (VRS) models distinguish pure technical efficiency from scale effects [25]. In the present study, CRS was retained as the primary specification to assess overall resource-use efficiency, while VRS was additionally applied to isolate pure technical efficiency and scale effects. Scale efficiency was subsequently derived from the ratio of CRS to VRS efficiency scores.

Although previous studies have assessed the efficiency of Greek public hospitals [7,12,18,26,27,28], limited evidence combines bias-corrected technical efficiency, scale-related effects, contextual determinants, and intertemporal productivity change within a common regional governance framework. The present study addresses this gap by integrating these complementary dimensions of hospital performance within a single analytical framework.

Against this background, the present study evaluates the efficiency and productivity of 13 Greek public hospitals over the period 2015–2021. The analytical framework combines input-oriented DEA under constant and variable returns to scale, bootstrap bias correction, exploratory Simar–Wilson bootstrap truncated regression and the Malmquist Productivity Index. Specifically, the study examines: (i) variation in overall and pure technical efficiency across hospitals; (ii) the contribution of scale efficiency to hospital performance; (iii) exploratory associations between selected contextual characteristics and hospital efficiency; and (iv) changes in hospital productivity over time. This integrated framework provides a multidimensional assessment of hospital performance within a common regional governance context.

## 2. Materials and Methods

### 2.1. Study Design, Data Sources, and Hospital Sample

The study setting encompassed 15 public hospitals operating under a Regional Health Authority in Greece. Of these, two hospitals were excluded. One hospital was excluded because its distinct legal and organizational status was considered to reflect a structurally different production environment and therefore to compromise comparability with the remaining hospitals. A second hospital reported no surgical activity during the study period and was excluded because its service-production profile was not considered sufficiently comparable with hospitals producing both DEA outputs. The analysis therefore included 13 hospitals, using annual data over the period 2015–2021.

Hospital-level operational, staffing, and financial data were obtained from the administrative database of the responsible Regional Health Authority following formal authorization for research purposes. The analysis was conducted at the hospital level, treating each hospital as a decision-making unit (DMU). No individual-level patient data or identifiable personal information were analyzed. To preserve institutional anonymity and confidentiality, all identifying details regarding the Regional Health Authority and participating hospitals were removed prior to analysis, and the hospitals were coded as F01-F13 throughout the study.

Municipal-level demographic and socioeconomic data for 2021 were obtained from the Hellenic Statistical Authority (ELSTAT), using population denominators from the 2021 Population and Housing Census. Hospital input and output data were also available for 2021, allowing the second-stage analysis to use each hospital’s 2021 efficiency estimate together with contemporaneous contextual variables. This ensured exact temporal alignment between the contextual characteristics and the efficiency outcome.

For the second-stage analysis, each hospital was geographically linked to the municipality in which it was physically located, and the corresponding 2021 municipal-level demographic and socioeconomic characteristics were assigned to that hospital. These measures were used as contextual proxies rather than as direct estimates of hospital catchment populations. Actual hospital service areas may extend beyond municipal boundaries, particularly for hospitals with broader referral roles; therefore, municipality-level characteristics should be interpreted as indicators of the local context in which each hospital operates rather than as precise measures of the population served.

### 2.2. Variable Measurement

The selection of DEA inputs, outputs, and contextual variables was guided by previous hospital-efficiency studies and by the availability and consistency of data across hospitals and years.

#### 2.2.1. DEA Inputs and Outputs

The selection of inputs and outputs is a critical stage in DEA because it defines the production process through which hospitals transform available resources into healthcare services [7]. In the present study, three inputs and two outputs were selected, guided by previous applications in the hospital sector and the availability of reliable data across all examined hospitals and years [7,29,30].

The three inputs were the number of operating beds, the number of personnel, and operating expenditure. Operating beds represented the available physical capacity of each hospital, while personnel were measured as total staff headcount across all professional categories (e.g., physicians, nursing staff, administrative and other personnel), as recorded in the administrative database. Because the included personnel were employed on a full-time basis, headcount corresponded numerically to full-time-equivalent staffing. Disaggregated annual staffing data by professional category were not consistently available and were therefore not incorporated into the DEA specification. Operating expenditure represented the non-labour financial resources used in hospital operations during the corresponding fiscal period.

The two outputs were the number of inpatients and the number of surgical procedures performed. These variables were selected to capture complementary dimensions of hospital service volume generated from the available physical, human, and financial resources. Because an input-oriented model was applied, the estimated efficiency scores indicate the proportional reduction in inputs that could be achieved while maintaining the observed level of outputs. As these outputs are volume-based and are not adjusted for case mix or quality, the resulting estimates are interpreted as volume-based technical efficiency within the specified production model rather than as comprehensive measures of overall hospital performance. With 13 hospitals, three inputs, and two outputs, the sample satisfied some commonly cited DEA dimensionality heuristics. Specifically, Golany and Roll [31] recommend that the number of DMUs should be at least twice the total number of inputs and outputs, whereas Dyson et al. [32] recommend at least twice the product of the number of inputs and outputs. These criteria correspond to minimum sample sizes of 10 and 12 DMUs, respectively, for the present specification. Nevertheless, the relatively small sample may still limit the discriminatory power of the DEA model and increase the likelihood of frontier saturation.

The isotonicity condition of the DEA specification was assessed using annual Spearman rank correlations between each of the three inputs and each of the two outputs. Positive input–output correlations were taken as evidence of monotonic consistency between resource use and service volume (Table 1); detailed annual coefficients are reported in Supplementary Table S1.

Operating expenditure was expressed in EUR and adjusted for inflation using the Gross Domestic Product (GDP) deflator, with 2021 as the base year. Annual GDP deflator data for Greece were obtained from the Organization for Economic Co-operation and Development (OECD) via FRED (series GRCGDPDEFAISMEI) [33], rebased to 2021 = 100, and used to express all operating expenditure figures in constant 2021 EUR. The deflator values used were: 2015 = 99.5, 2016 = 98.9, 2017 = 99.2, 2018 = 99.1, 2019 = 99.3, 2020 = 98.5, 2021 = 100.0.

#### 2.2.2. Environmental and Contextual Variables

Three contextual variables were included in the exploratory second-stage analysis: population ageing, population density, and unemployment. These variables were not included in the DEA production model because they do not represent resources directly consumed or services directly produced by hospitals; rather, they characterize the demographic and socioeconomic environment in which the hospitals operate. Given the limited number of hospital-level observations (*n* = 13), a parsimonious specification was adopted to reduce the risk of overfitting. Table 2 presents the operational definitions and measurement units of the contextual variables. Variables originally expressed as percentages were converted to proportions ranging from 0 to 1 for the second-stage analysis. Population density was divided by 1000 for regression and therefore entered the model in thousands of inhabitants per km^2^; descriptive statistics are reported in the original units.

### 2.3. Analytical Framework

The study followed a four-stage analytical framework combining annual input-oriented DEA under constant and variable returns to scale, bootstrap bias correction, exploratory Simar–Wilson bootstrap truncated regression, and the Malmquist Productivity Index. These methods were used, respectively, to estimate overall and pure technical efficiency and scale efficiency, correct potential bias in the DEA estimates, examine exploratory associations between selected 2021 contextual characteristics and hospital efficiency, and assess productivity change over the study period.

#### 2.3.1. Data Envelopment Analysis

Data Envelopment Analysis (DEA) is a non-parametric frontier method used to assess the relative efficiency of comparable decision-making units (DMUs) using multiple inputs and outputs. In this study, annual DEA models were estimated separately for each year from 2015 to 2021, with each of the 13 hospitals treated as a DMU using three inputs and two outputs.

An input-oriented model under the constant returns to scale (CRS) assumption was applied. The input orientation was selected because hospital administrators exercise greater operational control over resource inputs, such as beds, staffing, and operating expenditure, whereas demand for hospital services is comparatively less controllable. Accordingly, the model estimates the proportional reduction in inputs that could be achieved while maintaining the observed levels of inpatients and surgical procedures. The CRS specification was used to assess overall technical efficiency, which incorporates both pure technical and scale-related efficiency [17,24,25].

Under the input-oriented CRS specification, the efficiency of hospital *o* was estimated using the multiplier form of the CCR model. The model selects non-negative input and output weights that maximize the ratio of weighted outputs to weighted inputs for the hospital under evaluation, subject to the condition that the corresponding ratio does not exceed unity for any hospital in the sample.

$$\max h_{o}=\frac{\sum_{r=1}^{s}u_{r}y_{r0}}{\sum_{i=1}^{m}v_{i}x_{i0}}$$

$$\mathrm{subject}\;\mathrm{to}\;\frac{\sum_{r=1}^{s}u_{r}y_{rj}}{\sum_{i=1}^{m}v_{i}x_{ij}}\leq 1\forall j=1,\ldots ,n\;$$

$$u_{r},\;v_{i}\geq \varepsilon \;\forall r=1,\ldots ,s;\;i=1,\ldots ,\;m\;$$

where

h_0_ is the relative efficiency score of DMU_0_;

o is the DMU evaluated from the set of j = 1, …, *n* units;

j = the number of units, j = 1, …, *n*;

r = the number of outputs, r = 1, …, s;

i = the number of inputs, i = 1, …, m;

y_rj_ = the output amount of DMU j;

x_ij_ = the input amount of DMU j;

ε = a very small positive number (e.g., ε = 10^−6^);

u_r_, v_i_ = the coefficients for output r and input i, respectively, that maximize the objective function for the unit under examination each time.

To distinguish overall technical efficiency from scale-related effects, an input-oriented variable returns to scale (VRS) model was also estimated using the same inputs and outputs. The VRS specification provides an estimate of pure technical efficiency by allowing for variable returns to scale. Scale efficiency (SE) was calculated for each hospital and year as the ratio of CRS efficiency to VRS efficiency: $SE=\frac{{TE}_{CRS}}{{TE}_{VRS}}$.

Scale-efficiency scores range from 0 to 1, with a value of 1 indicating scale-efficient operation and values below 1 indicating that part of the observed overall technical inefficiency is attributable to scale inefficiency.

#### 2.3.2. Bootstrap Bias Correction

DEA efficiency estimates may be affected by sampling variation and upward bias because the efficiency frontier is constructed from the observed sample and the method does not explicitly distinguish inefficiency from statistical noise. To address this limitation, a smoothed homogeneous bootstrap procedure based on Simar and Wilson [34,35] was applied to the annual CRS efficiency estimates. Consistent with the implementation described by Tian et al. [36], 2000 bootstrap replications were generated and the efficiency scores were re-estimated in each replication. The estimated bias of the original efficiency score was calculated as:

$$\hat{Bias}\left({\hat{\theta }}_{ο}\right)=\;\frac{1}{Β}\sum_{b=1}^{Β}{\hat{\theta }}_{ο,b}^{*}-\;{\hat{\theta }}_{ο}$$

where ${\hat{\theta }}_{ο}$ is the original DEA efficiency estimate and ${\hat{\theta }}_{ο,b}^{*}$ is the corresponding estimate obtained from bootstrap replication b. The bias-corrected efficiency score was obtained as:

$${\hat{\theta }}_{ο,b}^{*}={\hat{\theta }}_{ο}-\;\hat{Bias}\left({\hat{\theta }}_{ο}\right)$$

The bootstrap procedure produced bias-corrected efficiency estimates and 95% confidence intervals for each hospital and year. These estimates were used as the primary efficiency measures in subsequent interpretation.

#### 2.3.3. Exploratory Second-Stage Bootstrap Truncated Regression

DEA estimates relative hospital efficiency using the selected inputs and outputs but does not directly examine whether contextual conditions are associated with efficiency. To examine exploratory associations between contextual characteristics and hospital efficiency, we assume the true relationship between efficiency and a hospital’s characteristics is given by:

$$\theta_{i}=Z_{i}\beta +\varepsilon_{i},\;i=1,\ldots ,n$$

where Z_i_ is the vector of contextual variables (including an intercept) for hospital i, expected to influence the efficiency score θ_i_ through the vector of parameters β, together with statistical noise ε_i_. Because θ_i_ is unobserved, we followed the the double-bootstrap procedure described as Algorithm 2 by Simar and Wilson [37,38]. The procedure first replaces the unobserved regress and θ_i_ with a bias-corrected estimate ${\hat{\theta }}_{i}^{bc}$, obtained via a bootstrap that re-estimates the DEA frontier from artificial data reflecting the assumed data-generating process. This bias-corrected estimate incorporates an empirical analogue of (3), given by:

$${\hat{\theta }}_{i}^{bc}\approx Z_{i}\beta +c_{i},\;i=1,\ldots ,n$$

where c_i_ ~ N (0, σ^2^) is assumed truncated, such that ${\hat{\theta }}_{i}^{bc}$ remains within the interval (0,1], corresponding to the theoretical support of an input-oriented DEA efficiency score. The model was estimated by maximum likelihood, following the input-oriented adaptation described by Badunenko and Tauchmann [38].

Inference for the resulting coefficients was then based on a separate parametric bootstrap with 2000 replications, in which truncated pseudo-observations were generated from the fitted model, the regression was re-estimated on each sample, and percentile-based confidence intervals were constructed from the resulting bootstrap distributions [37,38]. This bias-corrected estimate ${\hat{\theta }}_{i}^{bc}$, generated earlier in the procedure to produce the dependent variable for the regression, is distinct from the annual bias-corrected CRS-DEA estimates, which were obtained separately for each year (2015–2021) using a smoothed bootstrap procedure [34,35].

The second-stage analysis used each hospital’s 2021 bias-corrected DEA efficiency estimate as the dependent variable, temporally matched with contextual characteristics measured in 2021. Given the small sample (*n* = 13), the model was restricted to population ageing, population density, and unemployment, and the resulting coefficients were interpreted as exploratory associations rather than causal effects. To facilitate comparison of effect magnitudes across contextual variables measured on different scales, standardized coefficients for the primary specification (Model A1) were calculated by rescaling each estimated coefficient using the ratio of the predictor standard deviation to the standard deviation of the dependent variable, based on the same 13 hospital-level observations.

#### 2.3.4. Malmquist Productivity Index

The Malmquist Productivity Index (MPI) was calculated to assess year-over-year productivity changes over consecutive periods from 2015–2016 to 2020–2021. The MPI decomposes total factor productivity change into efficiency change (EC) and technological change (TC). Efficiency change indicates whether a hospital moves closer to or further away from the production frontier, whereas the technological change component reflects a shift in the frontier between two consecutive periods [39,40,41].

The input-oriented Malmquist Productivity Index compares each hospital’s performance relative to the production frontiers in periods (t) and (t+1). Following Färe et al. [42], the DEA-based Malmquist total factor productivity (TFP) index is expressed as follows:

$$M_{I}^{\;t+1}\left(y^{t+1},x^{t+1},y^{t},x^{t}\right)={\left[\frac{D_{I}^{t}\left(y^{t+1},x^{t+1}\right)}{D_{I}^{t}\left(y^{t},x^{t}\right)}\times \frac{D_{I}^{t+1}\left(y^{t+1},x^{t+1}\right)}{D_{I}^{t+1}\left(y^{t},x^{t}\right)}\right]}^{1/2}$$

where $M_{I}$ is the Malmquist index based on the input-oriented approach, $D_{I}$ is the input distance function, and x and y represent the input and output vectors, respectively. An input distance function indicates the degree to which the use of specific inputs can be reduced while keeping the output level constant, subject to the constraints of the production possibility set.

The Malmquist Productivity Index is decomposed into two components. The first is the Efficiency Change (EC) [42]:

$$EC=\frac{D_{I}^{t+1}\left(y^{t+1},x^{t+1}\right)}{D_{I}^{t}\left(y^{t},x^{t}\right)}$$

The second component is the Technological Change (TC), according to the following formula:

$$TC={\left[\frac{D_{I}^{t}\left(y^{t+1},x^{t+1}\right)}{D_{I}^{t+1}\left(y^{t+1},x^{t+1}\right)}\times \frac{D_{I}^{t}\left(y^{t},x^{t}\right)}{D_{I}^{t+1}\left(y^{t},x^{t}\right)}\right]}^{1/2}$$

Total Factor Productivity change is given by the product of EC and TC. An MPI value greater than 1 indicates productivity growth, a value below 1 indicates productivity decline, and a value equal to 1 indicates no change [43]. Hospital-level multi-period indices and annual aggregate indices across hospitals were summarized using geometric means, consistent with the multiplicative nature of Malmquist indices.

### 2.4. Software and Reproducibility

The CRS and VRS DEA efficiency estimates were obtained using PIM-DEA software, version 3.2 [44]. Bootstrap bias correction utilizing 2000 replications, the second-stage Simar–Wilson bootstrap truncated regression [37] and the Malmquist Productivity Index were estimated using R, version 4.5.2. A random seed of 123 was set to improve the reproducibility of the bootstrap results. Bootstrap bias correction was implemented using the *dea.boot()* function of the *Benchmarking* package [45,46], which applies the smoothed homogeneous bootstrap procedure of Simar and Wilson [34]. The smoothed bootstrap used the package-default bandwidth selection procedure, with bias correction performed on the input-distance scale and subsequently transformed to Farrell input-efficiency scores. Ninety-five percent confidence intervals for the bias-corrected efficiency estimates were constructed from the empirical distribution of the bootstrap replications at α = 0.05.

Data preprocessing and descriptive analyses were conducted in Microsoft Excel. The analytical dataset, variable definitions, computational code, and model specifications are available from the corresponding author upon reasonable request.

### 2.5. Ethical Considerations

The study used anonymized, aggregated hospital-level administrative data and involved no individual patients, identifiable personal information, or clinical interventions. Ethical review was therefore waived in accordance with the applicable institutional requirements, and informed consent was not applicable.

## 3. Results

### 3.1. Descriptive Analysis

Table 3 presents descriptive statistics for the average annual DEA inputs and outputs across the 13 hospitals over 2015–2021, with operating expenditure expressed in constant 2021 EUR. Considerable variation was observed in hospital size, resource use, and service activity. Average operating capacity was 209.95 beds per hospital, while mean staffing was 492.67 staff members. Mean annual operating expenditure was approximately EUR 6.36 million. On the output side, hospitals treated an average of 13,319.37 inpatients and performed 2795.38 surgical procedures annually. The wide range observed across several variables reflects substantial differences in hospital size and service profile.

Across the 13 hospital-level contextual observations used in the 2021 second-stage analysis, population ageing averaged 31.22% (SD 2.41; range 29.30–38.44%), population density averaged 3879.0 inhabitants/km^2^ (SD 7213.4; range 11.52–16,532.0), and unemployment averaged 17.38% (SD 1.64; range 15.75–22.33%).

Given the small sample size (*n* = 13) relative to the number of predictors, multicollinearity diagnostics were examined for the three contextual variables retained in the second-stage model. Table 4 presents the pairwise Pearson correlation matrix and variance inflation factors (VIFs) for population ageing, population density, and unemployment. All pairwise correlations were low (|r| ≤ 0.272), and VIF values ranged from 1.063 to 1.149, well below commonly used thresholds of concern (VIF > 5 or 10). These findings indicate that linear multicollinearity was not a material concern in the second-stage analysis and did not warrant exclusion of any of the three predictors from the primary specification (Model A1).

Separately, the isotonicity condition of the DEA specification was examined using annual Spearman rank correlations between each of the three DEA inputs and each of the two outputs. All 42 input–output correlations were positive (ρ = 0.423–0.945), indicating relationships consistent with the isotonicity condition across the annual production sets. Although some correlations involving surgical procedures were not statistically significant in individual years, none were negative (Supplementary Table S1).

### 3.2. DEA and Bootstrap-Corrected Efficiency

Table 5 presents the conventional input-oriented CRS-DEA efficiency scores for the 13 hospitals over 2015–2021. Mean CRS efficiency ranged from 0.8324 in 2018 to 0.9130 in 2021, indicating year-to-year variation in relative resource-use efficiency. Hospitals F01 and F03 remained on the estimated CRS efficiency frontier throughout the study period, achieving an efficiency score of 1 in every year. Hospitals F06, F08, F09, F10, and F11 also reached the estimated frontier in one or more individual years. Overall, the number of hospitals located on the CRS frontier ranged from 4 to 5 per year, corresponding to 30.8% of the sample in 2015, 2016, 2018, and 2020, and 38.5% in 2017, 2019, and 2021. In contrast, F12 and F13 generally recorded lower efficiency scores over much of the study period. The lowest conventional CRS efficiency score was observed for F12 in 2020 (0.4877).

Table 6 presents the scale-efficiency scores for the 13 hospitals over 2015–2021, calculated as the ratio of CRS to VRS efficiency scores. The corresponding VRS pure technical efficiency scores are reported in Supplementary Table S2. Mean scale efficiency ranged from 0.8981 in 2020 to 0.9416 in 2021, indicating variation in the extent to which scale effects contributed to overall technical inefficiency across years. Hospitals F01 and F03 were scale efficient relative to the observed sample throughout the study period, achieving a scale-efficiency score of 1 in every year. Hospitals F06, F08, F09, F10, and F11 also achieved scale efficiency in one or more individual years. Overall, the number of scale-efficient hospitals ranged from 4 to 5 per year, corresponding to 30.8% of the sample in 2015, 2016, 2018, and 2020, and 38.5% in 2017, 2019, and 2021. F12 generally recorded the lowest scale-efficiency scores, while F13 showed comparatively lower scale efficiency in several years but improved by 2021. The lowest scale-efficiency score was observed for F12 in 2020 (0.5373).

Because conventional DEA scores may be affected by finite-sample bias, bootstrap correction was applied to the CRS efficiency estimates. The resulting bias-corrected efficiency scores and corresponding 95% bootstrap confidence intervals are presented in Table 7. Mean bias-corrected efficiency ranged from 0.7486 in 2020 to 0.8556 in 2021. Across all years, the bias-corrected estimates were lower than the corresponding conventional CRS-DEA scores, with an average difference of approximately 0.074, indicating the magnitude of the upward bias in the uncorrected estimates. No hospital achieved a bias-corrected efficiency score of 1, and variation in efficiency remained evident across hospitals and years.

After bootstrap correction, no hospital achieved an efficiency score of 1, and variation in technical efficiency remained evident across hospitals and years. The highest numerical bias-corrected estimate over the study period was 0.955 for F10 in 2021, whereas the lowest was 0.444 for F12 in 2020. In 2021, F10 recorded the highest numerical estimate (0.955), followed by F06 (0.919) and F04 (0.913), while F12 recorded the lowest estimate (0.669). However, bootstrap confidence intervals overlapped for several hospital-year comparisons; therefore, these numerical differences should not be interpreted as definitive efficiency rankings.

### 3.3. Second-Stage Truncated Regression with Double-Bootstrap

Table 8 presents the results of the exploratory second-stage truncated regression estimated using the double-bootstrap procedure described as Algorithm 2 by Simar and Wilson [37], with 2000 bootstrap replications. The dependent variable was the bootstrap bias-corrected CRS-DEA efficiency estimate. Pairwise Pearson correlations and variance inflation factors (VIF) were computed for the three contextual predictors. Model A1 included all three retained predictors: population ageing, population density, and unemployment. Given the small sample size (*n* = 13) relative to the number of predictors, and to guard against multicollinearity and overfitting, three additional sensitivity specifications (Models A2–A4) were estimated, each excluding one predictor in turn, to assess the stability of the coefficient estimates across alternative model specifications. Full results for all specifications, including bootstrap standard errors, z-values, *p*-values, and 95% bootstrap confidence intervals, are reported in Supplementary Tables S3–S6.

In the primary specification (Model A1), all three contextual variables were significantly associated with the bias-corrected efficiency estimate. Population ageing was negatively associated with efficiency (β = −2.664, *p* < 0.001), as were population density (β = −0.0099, *p* < 0.001) and unemployment (β = −3.069, *p* < 0.001). The corresponding standardized coefficients were −0.662 for population ageing, −0.734 for population density, and −0.520 for unemployment. In absolute terms, population density had the largest standardized coefficient, followed by population ageing and unemployment. These comparisons are intended only to facilitate comparison across predictors measured on different scales and should be interpreted cautiously given the small sample size and exploratory nature of the second-stage analysis.

The sensitivity analyses (Models A2–A4) showed that the direction of the estimated associations remained broadly consistent across alternative specifications. The coefficient for population ageing remained negative and statistically significant when population density was excluded (Model A2: β = −2.281, *p* < 0.001) and when unemployment was excluded (Model A4: β = −2.714, *p* < 0.001). Population density also remained negatively associated with efficiency when population ageing was excluded (Model A3: β = −0.0071, *p* < 0.05). Similarly, unemployment retained a negative association in both reduced specifications in which it was included, although the level of statistical significance was attenuated relative to the primary model (Model A2: β = −2.498 *p* < 0.05; Model A3: β = −2.920, *p* < 0.05). Overall, the consistency in coefficient direction across specifications indicates stability in the direction of the observed associations across the reduced specifications. Nevertheless, given the small sample size (*n* = 13) and the exploratory nature of the second-stage analysis, these findings should be interpreted cautiously as associations rather than causal effects.

### 3.4. Malmquist Productivity Analysis

Table 9 presents the MPI and its decomposition into EC and TC for the 13 hospitals over 2015–2021. Hospital-level multi-period values were summarized using geometric means. The average MPI was below 1 for all hospitals, indicating an overall decline in productivity over the study period, although the magnitude of the decline varied across hospitals.

EC values were generally closer to 1 than TC, suggesting comparatively modest changes in hospitals’ relative positions with respect to the estimated frontier. Hospital-level EC values ranged from 0.9829 for F12 to 1.085 for F04. Six hospitals (F04, F05, F07, F08, F11, and F13) recorded EC values above 1, indicating movement toward the estimated frontier, whereas others recorded values below 1. F01 and F03 recorded EC values equal to 1, indicating no change in relative efficiency. By contrast, F02, F09, F10, and F12 recorded EC values below 1, indicating a deterioration in relative efficiency.

Technological Change (TC) was below 1 for all hospitals, ranging from 0.8374 for F06 to 0.9159 for F01. This consistent pattern indicates an inward shift in the estimated production frontier over the study period. Consequently, the observed decline in total factor productivity was primarily driven by technological change, whereas efficiency change remained comparatively stable. The Malmquist Index was below 1 for all hospitals, indicating that none experienced an average increase in productivity over the 2015–2021 period.

Substantial year-to-year heterogeneity was nevertheless observed. Productivity declined markedly during 2015–2016 (MPI = 0.6779; −32.21%) and 2019–2020 (MPI = 0.7802; −21.98%). In contrast, productivity increased slightly during 2016–2017 (MPI = 1.0028; +0.28%) and again during 2020–2021 (MPI = 1.0234; +2.34%). Productivity remained approximately stable during 2017–2018 (MPI = 0.9888; −1.12%) and declined moderately during 2018–2019 (MPI = 0.9503; −4.97%). Thus, although the overall multi-period pattern indicated productivity contraction, this aggregate result masked considerable variation across consecutive annual periods.

## 4. Discussion

The present study provides a multidimensional assessment of volume-based technical efficiency and productivity among 13 public hospitals operating within a Regional Health Authority in Greece. The principal findings were fourfold. First, conventional CRS efficiency varied across hospitals and years, while bootstrap correction produced systematically lower and more conservative estimates. Second, the VRS sensitivity analysis indicated that scale-related effects contributed to overall technical inefficiency, although their magnitude differed across hospitals and years. Third, the exploratory Simar–Wilson second-stage analysis identified negative associations between the 2021 bias-corrected efficiency estimates and population ageing, population density, and unemployment. Finally, the Malmquist analysis indicated an overall contraction in productivity over 2015–2021, driven predominantly by an inward shift in the estimated production frontier, but with substantial year-to-year heterogeneity.

Annual mean bias-corrected CRS efficiency ranged from approximately 0.75 to 0.86, indicating that the hospitals generally operated below the estimated production frontier. These estimates are broadly consistent with previous evidence from Greek public hospitals, although direct comparisons should be made cautiously because studies differ in hospital samples, input–output specifications, time periods, and assumptions regarding returns to scale. Dimas et al. [26] examined 22 Greek public general hospitals during 2003–2005 and reported a mean efficiency score of approximately 0.85. Kalogeropoulou et al. [47] estimated a mean efficiency score of 0.889 for 26 public hospitals in 2009, while Xenos et al. [48], using data from the same year, reported mean efficiency scores of 0.875 when a quality indicator was included and 0.895 when it was excluded. Katharakis et al. [18] estimated a mean efficiency score of approximately 0.816. More recently, Fourlopoulou et al. [27], using a substantially larger national sample and a cost-based Malmquist approach, also identified important variation in the performance of Greek public hospitals over a period overlapping with the present study. Taken together, these findings suggest persistent scope for more efficient resource use within Greek public hospitals, while also demonstrating the sensitivity of DEA estimates to model specification and the composition of the comparison set. The bootstrap results reinforce the need for caution when interpreting conventional DEA scores. Bias correction reduced the efficiency estimates by approximately 0.074 on average, and no hospital retained a bias-corrected efficiency score of 1. This finding is consistent with the finite-sample properties of DEA, whereby an empirical frontier constructed from a limited number of observations may overestimate efficiency [34]. A similar effect of bootstrap adjustment has been reported in previous hospital-efficiency research by Katharakis et al. [18]. Moreover, the overlap of bootstrap confidence intervals across several hospital-year comparisons indicates that numerical differences between hospitals should not be interpreted as definitive performance rankings. DEA identifies relative performance within the observed production set rather than an absolute or externally validated standard of best practice [24,32].

The CRS–VRS comparison provides additional insight into the source of observed inefficiency. Mean scale efficiency remained relatively high, ranging from approximately 0.90 to 0.94 across years, but values below unity indicate that scale-related factors contributed to the gap between overall CRS efficiency and pure technical efficiency for some hospitals. This distinction is important because inefficiency under CRS cannot automatically be attributed to managerial or operational performance alone; part may arise from the scale at which a hospital operates [25]. Previous evidence from Greek public hospitals has similarly demonstrated the relevance of distinguishing technical, pure technical, and scale efficiency [29,49]. Consequently, improvement strategies should distinguish between inefficiency potentially amenable to changes in internal resource use and inefficiency associated with hospital scale, service configuration, or broader system organization. Recent Greek evidence further suggests that policy interventions may affect these dimensions differently, with changes in resource use, organizational arrangements, and cost-containment measures influencing hospital efficiency in distinct ways [49].

The second-stage findings should be interpreted separately from the DEA and Malmquist results. The Simar–Wilson analysis was exploratory, based on only 13 hospital-level observations, and examined contemporaneous associations between 2021 efficiency estimates and three municipal-level contextual characteristics. Although the direction of the coefficients remained broadly stable across the sensitivity specifications, these associations should not be interpreted as causal effects. Rather, they indicate that the local demographic and socioeconomic environment may be relevant to observed differences in volume-based technical efficiency. Previous studies have similarly identified contextual characteristics as potentially important determinants of hospital efficiency [19,43,50].

Population ageing was negatively associated with the bias-corrected efficiency estimate. Demographic structure may influence hospital performance through differences in healthcare need, utilization patterns, and the resource intensity of care [43]. Older populations may generate greater demand for hospital services and place additional pressure on healthcare resources [43,51,52]. In a volume-based DEA framework, this may translate into greater resource use without a proportionate increase in the measured number of inpatient episodes or surgical procedures. This interpretation remains tentative, particularly because municipal population ageing was used as a contextual proxy and does not necessarily represent the demographic composition of each hospital’s actual catchment population.

Population density was also negatively associated with efficiency. Population density has been identified as an important contextual determinant of healthcare resource-use efficiency, although both the direction and magnitude of its association appear to vary across settings [23,53,54]. Notably, recent evidence from Croatian hospitals reported a negative association between population density and technical efficiency using a Simar–Wilson second-stage model [54], while Xing et al. similarly identified a negative effect of population density on operational efficiency [53]. Hospitals located in densely populated areas may face greater service demand, congestion, capacity pressures, and more complex patterns of patient flow. Under such circumstances, lower measured efficiency need not necessarily indicate weaker managerial performance; it may partly reflect the resource requirements associated with maintaining access and responding to greater population demand. This interpretation also raises a potential tension between efficiency and equity, since resource requirements may vary according to population concentration, geographical accessibility, and local healthcare needs [13]. The finding should therefore be interpreted in conjunction with population need and accessibility rather than as evidence that densely populated areas are intrinsically less efficient.

Unemployment showed a similarly negative association with efficiency. Socioeconomic conditions can influence both healthcare utilization and the capacity of populations to access alternative forms of care [8,13]. Recent evidence also suggests that unemployed and socioeconomically disadvantaged populations may experience higher levels of unmet healthcare need [55]. Such conditions may increase reliance on publicly funded healthcare services and may contribute to delayed access to care, potentially increasing the resource requirements associated with hospital treatment. In a volume-based DEA framework, these additional resource requirements may not be accompanied by proportional increases in the measured outputs. Importantly, Karakolias and Polyzos [13] highlighted persistent inequalities in resource allocation and funding under decentralized governance in Greece, reinforcing the need to interpret hospital efficiency within its regional socioeconomic context rather than independently of population need.

The Malmquist results add a dynamic perspective to the static efficiency estimates. Overall productivity declined over 2015–2021, but this aggregate pattern concealed substantial year-to-year variation. Productivity increased during 2016–2017, remained approximately stable during 2017–2018, declined during 2018–2019, fell markedly during 2019–2020, and subsequently recovered during 2020–2021. The overall decline was driven predominantly by the Malmquist technological-change component rather than by a systematic deterioration in hospitals’ relative efficiency. This component should be interpreted as a shift in the estimated production frontier and not narrowly as the adoption or deterioration of medical technology [41,42]. In hospital settings, such frontier shifts may reflect broader changes in resource availability, infrastructure, workforce capacity, organizational arrangements, and external system-level shocks [9,27].

The pronounced productivity contraction during 2019–2020 coincided with the onset of the COVID-19 pandemic and should therefore be interpreted within this exceptional period of system-wide disruption rather than as evidence of technological deterioration in the conventional sense. Similar pandemic-related pressures on the efficiency of Greek public hospitals have been reported by Mitakos and Mpogiatzidis [56], who identified scale inefficiencies under the exceptional operating conditions of 2020. Fourlopoulou et al. [27] likewise reported a substantial decline in the productivity of Greek public hospitals during the pandemic period, driven primarily by deterioration of the estimated cost frontier under conditions of increased healthcare demand and strained resources. Their findings also indicated signs of recovery following the lifting of strict pandemic restrictions [27]. The subsequent improvement observed in the present study during 2020–2021 is also compatible with partial adaptation and recovery following the initial disruption, a pattern also observed in recent evidence from Greek public hospitals [28], although the observational design does not allow either the decline or the recovery to be attributed directly to COVID-19. More broadly, the study period should be considered in the context of persistent financial constraints, workforce pressures, infrastructure limitations, and regional inequalities within the Greek health system [9,10,13,57]. These system-level conditions provide a plausible context for shifts in the estimated production frontier but cannot be individually identified as causal drivers by the Malmquist decomposition [41,42].

From a policy and managerial perspective, the findings support the use of DEA as a tool for structured regional performance review and decision support rather than as a mechanism for mechanically ranking hospitals or imposing uniform resource-reduction targets [3,5,58]. Hospitals with comparatively high or improving efficiency may provide useful statistical reference points for investigating differences in resource organization and service delivery [58,59]. However, hospitals located on the estimated DEA frontier should not automatically be regarded as validated best-practice institutions, because DEA identifies relative performance within the specific production set under the selected model specification [24,30,58]. Moreover, the present model does not adjust for patient case-mix complexity, quality of care, geographical accessibility, or specialization, all of which may influence observed efficiency differences [4,30,59]. Regional Health Authorities could therefore use efficiency analysis as an initial screening and decision-support instrument to identify hospitals warranting closer operational investigation, while interpreting DEA results alongside quality-of-care indicators, population need, accessibility, staffing conditions, and local service responsibilities.

### Limitations and Future Research

This study has several limitations. It included 13 public hospitals from a single Regional Health Authority. Although the sample met commonly used DEA dimensionality criteria, the small number of hospitals may reduce discriminatory power and limits generalizability [30,31]. Because DEA is sample-specific, efficient hospitals should be interpreted as relative statistical peers rather than validated best-practice institutions. Future studies should include larger samples across multiple Greek Health Regions.

Second, the DEA outputs captured service volume and were not adjusted for case mix or quality. Hospitals treating more complex patients or using additional resources to achieve higher-quality outcomes may therefore appear less efficient, while high-volume hospitals may appear more efficient when quality differences are not captured [4,30]. The magnitude of this potential bias cannot be quantified with the available data. Future studies should incorporate case-mix-adjusted activity, quality indicators, and more detailed workforce measures.

Finally, the Simar–Wilson second-stage analysis was based on only 13 observations and should therefore be interpreted as exploratory rather than causal. Municipality-level variables were used as contextual proxies and may not fully represent hospital catchment populations. In addition, the Malmquist technological-change component reflects shifts in the estimated production frontier but cannot identify their underlying causes. Future research should use larger longitudinal datasets, patient-flow-based catchment areas, and direct measures of workforce, infrastructure, digitalization, and organizational change, while integrating efficiency with quality, accessibility, and population need.

## 5. Conclusions

This study identified substantial variation in volume-based technical efficiency among 13 public hospitals during 2015–2021. Bootstrap correction produced more conservative efficiency estimates than conventional DEA, while the VRS analysis indicated that scale-related factors contributed to inefficiency in some hospitals. The exploratory Simar–Wilson analysis suggested negative associations between efficiency and population ageing, population density, and unemployment, although these findings should not be interpreted causally. The Malmquist analysis showed an overall decline in productivity over the study period, driven mainly by shifts in the estimated production frontier and marked by substantial year-to-year variation, including a pronounced contraction during 2019–2020.

For Regional Health Authorities, DEA can support structured performance review by identifying hospitals that warrant closer operational investigation and statistical peers from which organizational practices may be explored. However, efficiency scores should not be used as stand-alone rankings or uniform resource-reduction targets. Performance assessment should combine efficiency with case mix, quality of care, accessibility, population need, and local service responsibilities. Future research should extend the analysis across multiple Greek Health Regions and develop integrated efficiency–quality frameworks with case-mix- and accessibility-adjusted performance targets.

## Figures and Tables

**Table 1:** Input and output variables used in the Data Envelopment Analysis.

Variable	Role	Operational Measurement	Unit
Operating beds	Input	Number of beds available for hospital operation	Number
Personnel	Input	Total hospital staff headcount across all professional categories (numerically equivalent to FTE staffing)	Number
Operating expenditure	Input	Non-labor operating expenses incurred during the corresponding fiscal year	EUR
Inpatients	Output	Total number of inpatients during the year	Number
Surgical procedures	Output	Total number of surgical procedures performed during the year	Number

**Table 2:** Contextual variables included in the exploratory second-stage analysis.

Variable (Symbol)	Operational Definition	Measurement/Coding
Population ageing (A)	Residents aged ≥65 years as a percentage of the total municipal population	Percentage (%) converted to proportion (0–1) for regression
Population density (D)	Municipal population divided by geographical area	Inhabitants/km^2^ divided by 1000 for regression
Unemployment (U)	Unemployed residents as a percentage of the total municipal population	Percentage (%) converted to proportion (0–1) for regression

**Table 3:** Descriptive Statistics of Average Annual DEA Inputs and Outputs, 2015–2021.

Variable	Mean	SD	Minimum	Maximum
Operating beds	209.95	139.99	85	682
Personnel	492.67	297.05	195	1556
Operating expenditure	6,361,551.02	9,176,267.03	794,621	45,117,015
Inpatients	13,319.37	12,313.20	3776	57,828
Surgical procedures	2795.38	2324.28	627	10,551

**Table 4:** Pearson correlation matrix and variance inflation factors (VIFs) for contextual predictors included in the second-stage analysis.

	Population Ageing	Population Density	Unemployment	VIF	1/VIF
Population ageing	1	−0.272	−0.01	1.087	0.9199
Population density	−0.272	1	−0.232	1.149	0.8703
Unemployment	−0.01	−0.232	1	1.063	0.9407

**Table 5:** Input-oriented CRS-DEA efficiency scores, 2015–2021.

DMU	2015	2016	2017	2018	2019	2020	2021
F01	1	1	1	1	1	1	1
F02	0.8394	0.7452	0.7429	0.7006	0.7281	0.7684	0.8045
F03	1	1	1	1	1	1	1
F04	0.6122	0.6613	0.7325	0.7376	0.7228	0.7143	1
F05	0.7985	0.8014	0.8153	0.7213	0.6883	0.7766	0.9278
F06	1	1	0.9527	0.9575	1	0.792	1
F07	0.8552	0.9114	0.8542	0.716	0.7638	0.8059	0.9228
F08	0.8142	0.8408	0.7916	0.8323	0.9584	1	1
F09	0.8858	0.8583	1	0.9438	1	1	0.8331
F10	1	1	1	1	0.8595	0.8951	0.9904
F11	0.7599	0.7392	1	1	1	0.9522	0.7808
F12	0.7723	0.6801	0.6671	0.6706	0.597	0.4877	0.6966
F13	0.6797	0.7698	0.659	0.5413	0.5184	0.6494	0.9131
Mean	0.8475	0.8467	0.8627	0.8324	0.8336	0.8340	0.9130
Median	0.8394	0.8408	0.8542	0.8323	0.8595	0.8059	0.9278
Maximum	1	1	1	1	1	1	1
Minimum	0.6122	0.6613	0.6590	0.5413	0.5184	0.4877	0.6966
St. Deviation	0.1225	0.1210	0.1303	0.1528	0.1663	0.1535	0.0987
Frontier hospitals, *n* (%)	4 (30.8%)	4 (30.8%)	5 (38.5%)	4 (30.8%)	5 (38.5%)	4 (30.8%)	5 (38.5%)

**Table 6:** Scale Efficiency Scores derived from CRS and VRS DEA, 2015–2021.

DMU	2015	2016	2017	2018	2019	2020	2021
F01	1	1	1	1	1	1	1
F02	0.9058	0.9217	0.9691	0.9937	0.9896	0.9568	0.9991
F03	1	1	1	1	1	1	1
F04	0.8064	0.7611	0.7591	0.7936	0.8214	0.9167	1
F05	0.9226	0.9903	0.9995	0.9552	0.9421	0.947	0.9737
F06	1	1	0.9527	0.9575	1	0.792	1
F07	0.9465	0.9759	0.99	0.9442	0.9845	0.8678	0.9228
F08	0.976	0.9932	0.9895	0.9876	0.9984	1	1
F09	0.9315	0.9281	1	0.9927	1	1	0.9579
F10	1	1	1	1	0.9889	0.91	0.9904
F11	0.7599	0.7392	1	1	1	0.9522	0.7808
F12	0.7723	0.6801	0.6818	0.6706	0.6628	0.5373	0.703
F13	0.7243	0.7698	0.6853	0.5812	0.7098	0.7961	0.9131
Mean	0.9035	0.9046	0.9252	0.9136	0.9306	0.8981	0.9416
Median	0.9315	0.9759	0.9900	0.9876	0.9896	0.9470	0.9904
Maximum	1	1	1	1	1	1	1
Minimum	0.7243	0.6801	0.6818	0.5812	0.6628	0.5373	0.7030
St. Deviation	0.0980	0.1157	0.1205	0.1349	0.1147	0.1251	0.0911
Frontier hospitals, *n* (%)	4 (30.8%)	4 (30.8%)	5 (38.5%)	4 (30.8%)	5 (38.5%)	4 (30.8%)	5 (38.5%)

**Table 7:** Bias-Corrected CRS-DEA Efficiency Scores with Bootstrap 95% Confidence Intervals, 2015–2021.

DMU	2015	2016	2017	2018	2019	2020	2021
F01	0.886 [0.811–0.995]	0.889 [0.807–0.994]	0.885 [0.795–0.995]	0.865 [0.779–0.993]	0.855 [0.759–0.993]	0.854 [0.744–0.992]	0.907 [0.766–0.997]
F02	0.760 [0.677–0.833]	0.677 [0.604–0.740]	0.686 [0.620–0.738]	0.638 [0.563–0.696]	0.659 [0.565–0.721]	0.687 [0.595–0.762]	0.765 [0.698–0.801]
F03	0.861 [0.721–0.991]	0.859 [0.738–0.993]	0.866 [0.733–0.995]	0.842 [0.686–0.993]	0.836 [0.676–0.991]	0.844 [0.669–0.992]	0.906 [0.763–0.997]
F04	0.561 [0.499–0.610]	0.614 [0.558–0.657]	0.692 [0.645–0.729]	0.702 [0.652–0.734]	0.690 [0.645–0.719]	0.658 [0.609–0.709]	0.913 [0.815–0.997]
F05	0.735 [0.673–0.792]	0.740 [0.678–0.796]	0.763 [0.692–0.811]	0.657 [0.585–0.717]	0.621 [0.548–0.683]	0.716 [0.649–0.770]	0.896 [0.858–0.924]
F06	0.880 [0.791–0.993]	0.901 [0.825–0.992]	0.896 [0.835–0.948]	0.903 [0.831–0.952]	0.938 [0.873–0.994]	0.736 [0.683–0.784]	0.919 [0.823–0.997]
F07	0.804 [0.750–0.849]	0.848 [0.784–0.905]	0.808 [0.759–0.849]	0.668 [0.613–0.712]	0.717 [0.659–0.758]	0.735 [0.674–0.798]	0.877 [0.813–0.920]
F08	0.765 [0.710–0.808]	0.788 [0.738–0.834]	0.752 [0.707–0.786]	0.779 [0.724–0.824]	0.912 [0.853–0.952]	0.875 [0.793–0.990]	0.908 [0.798–0.996]
F09	0.813 [0.737–0.879]	0.796 [0.730–0.851]	0.875 [0.765–0.993]	0.890 [0.820–0.937]	0.938 [0.867–0.991]	0.897 [0.801–0.991]	0.805 [0.773–0.830]
F10	0.927 [0.835–0.994]	0.895 [0.821–0.992]	0.882 [0.778–0.993]	0.886 [0.802–0.992]	0.805 [0.749–0.851]	0.831 [0.772–0.886]	0.955 [0.913–0.987]
F11	0.718 [0.666–0.755]	0.677 [0.617–0.733]	0.912 [0.794–0.993]	0.870 [0.791–0.990]	0.870 [0.788–0.991]	0.870 [0.771–0.948]	0.736 [0.668–0.777]
F12	0.721 [0.668–0.768]	0.629 [0.573–0.676]	0.628 [0.573–0.665]	0.616 [0.559–0.666]	0.541 [0.491–0.593]	0.444 [0.400–0.484]	0.669 [0.635–0.695]
F13	0.632 [0.583–0.675]	0.709 [0.650–0.765]	0.620 [0.573–0.655]	0.502 [0.457–0.537]	0.480 [0.441–0.514]	0.586 [0.531–0.642]	0.868 [0.808–0.910]
Mean	0.7740	0.771	0.7897	0.7553	0.7585	0.7486	0.8556
Median	0.7648	0.7883	0.808	0.7794	0.8052	0.7364	0.8958
Maximum	0.9267	0.9008	0.9125	0.9033	0.938	0.8968	0.9548
Minimum	0.5613	0.6135	0.6198	0.502	0.4801	0.4437	0.6692
St. Deviation	0.1001	0.0993	0.1019	0.1264	0.1462	0.1278	0.0818

Note: Values are presented as bias-corrected efficiency scores, with bootstrap confidence intervals in square brackets.

**Table 8:** Exploratory Second-Stage Simar–Wilson Truncated Regression Results with Double Bootstrap across Model Specifications.

Variable	Model A1	Std. Coefficient (A1)	Model A2	Model A3	Model A4
Population ageing	−2.6641 ***	−0.662	−2.2811 ***	-	−2.7135 ***
Population density	−0.0099 ***	−0.734	-	−0.0071 **	−0.0079 ***
Unemployment	−3.0686 ***	−0.520	−2.4984 ***	−2.9197 **	-

Note: ** and *** indicate statistical significance at the 10%, 5% and 1% levels, respectively. Standardized coefficients are reported for Model A1 and were obtained by rescaling each coefficient using the ratio of the predictor standard deviation to the dependent-variable standard deviation.

**Table 9:** Malmquist Productivity Indices by Hospital and Annual Productivity Change, 2015–2021.

DMU/Period	Efficiency Change (EC)	Technological Change (TC)	Malmquist Productivity Index (MPI)	Percentage Change in Productivity
F01	1	0.9159	0.9159	−8.41%
F02	0.9929	0.8623	0.8562	−14.38%
F03	1	0.8823	0.8823	−11.77%
F04	1.0852	0.8508	0.9233	−7.67%
F05	1.0253	0.8930	0.9157	−8.43%
F06	1	0.8374	0.8374	−16.26%
F07	1.0128	0.8697	0.8808	−11.92%
F08	1.0349	0.8694	0.8997	−10.03%
F09	0.9898	0.8397	0.8312	−16.88%
F10	0.9984	0.9134	0.9119	−8.81%
F11	1.0045	0.8807	0.8847	−11.53%
F12	0.9829	0.8815	0.8664	−13.36%
F13	1.0504	0.8795	0.9239	−7.61%
2015–2016	1.0019	0.6776	0.6779	−32.21%
2016–2017	1.0237	0.9781	1.0028	0.28%
2017–2018	0.9615	1.029	0.9888	−1.12%
2018–2019	1.0003	0.9504	0.9503	−4.97%
2019–2020	1.0097	0.7725	0.7802	−21.98%
2020–2021	1.1264	0.9075	1.0234	2.34%

## Data Availability

The data supporting the findings of this study were obtained from the administrative database of the Regional Health Authority under specific authorization for the present research. Due to institutional confidentiality agreements and privacy restrictions, the raw administrative data are not publicly available but may be made available from the corresponding author upon reasonable request and subject to permission from the granting authority.

## Supplementary Materials

The following supporting information can be downloaded at: https://www.mdpi.com/article/10.3390/healthcare14183084/s1, Table S1. Annual Spearman rank correlations between DEA inputs and outputs, 2015–2021; Table S2. DEA efficiency scores under variable returns to scale (VRS), 2015–2021; Tables S3–S6. Exploratory second-stage Simar–Wilson truncated regression results with double bootstrap for Models A1–A4.

## Abbreviations

The following abbreviations are used in this manuscript:

CRS	Constant Returns to scale
COVID-19	Coronavirus disease 19
DEA	Data Envelopment Analysis
DMU	Decision-making unit
EC	Efficiency Change
EUR	Euro
GDP	Gross Domestic Product
MPI	Malmquist Productivity Index
OECD	Organisation for Economic Co-operation and Development
TC	Technological Change
TFP	Total Factor Productivity
VRS	Variable returns to scale
