# Supplementary material for: Evaluating Hospital Efficiency and Productivity: A Bootstrap-Corrected DEA and Malmquist Analysis
Source: Healthcare (Basel). 2026 Sep 19;14(18):3084. doi: 10.3390/healthcare14183084 (PMC13606170; doi:10.3390/healthcare14183084)
Supplement: Supplementary file 1 [file healthcare-14-03084-s001.zip › healthcare-4528402-supplementary.pdf]

Article

Evaluating Hospital Efficiency and Productivity: A Bootstrap-Corrected DEA and Malmquist Analysis

Supplementary Materials

Table S1. Annual Spearman rank correlations between DEA inputs and outputs, 2015–2021

Year	Operating Beds–Inpatients ϱ	p-value	Operating Beds–Surgical procedures ϱ	p-value	Personnel–Inpatients ϱ	p-value	Personnel–Surgical procedures ϱ	p-value	Operating Expenditures–Inpatients ϱ	p-value	Operating Expenditures–Surgical procedures ϱ	p-value
**2015**	0,945	0,000	0,549	0,052	0,879	0,000	0,582	0,037	0,879	0,000	0,665	0,013
**2016**	0,907	0,000	0,670	0,012	0,872	0,000	0,680	0,011	0,830	0,000	0,489	0,090
**2017**	0,912	0,000	0,714	0,006	0,857	0,000	0,742	0,004	0,916	0,000	0,795	0,001
**2018**	0,861	0,000	0,677	0,011	0,852	0,000	0,648	0,017	0,912	0,000	0,714	0,006
**2019**	0,846	0,000	0,692	0,009	0,916	0,000	0,795	0,001	0,874	0,000	0,742	0,004
**2020**	0,841	0,000	0,731	0,005	0,923	0,000	0,874	0,000	0,786	0,001	0,692	0,009
**2021**	0,896	0,000	0,423	0,150	0,698	0,008	0,440	0,133	0,824	0,001	0,423	0,150

Note: ϱ denotes Spearman's rank correlation coefficient. All 42 annual input-output correlations were positive (range 0.423–0.945). Positive correlations support the isotonicity condition of the DEA specification. Statistical significance is reported for transparency but is not the sole criterion for isotonicity.

Table S2. DEA efficiency scores under variable returns to scale (VRS), 2015–2021

DMU	2015	2016	2017	2018	2019	2020	2021
F01	1	1	1	1	1	1	1
F02	0.9267	0.8085	0.7666	0.7051	0.7358	0.8031	0.8052
F03	1	1	1	1	1	1	1
F04	0.7591	0.8689	0.965	0.9294	0.8799	0.7792	1
F05	0.8655	0.8093	0.8157	0.7551	0.7306	0.82	0.9529
F06	1	1	1	1	1	1	1
F07	0.9035	0.9338	0.8628	0.7583	0.7758	0.9287	1
F08	0.8342	0.8466	0.8	0.8427	0.96	1	1
F09	0.951	0.9248	1	0.9508	1	1	0.8697
F10	1	1	1	1	0.8692	0.9836	1
F11	1	1	1	1	1	1	1
F12	1	1	0.9784	1	0.9007	0.9077	0.9909
F13	0.9385	1	0.9616	0.9314	0.7303	0.8157	1
Mean	0.9368	0.9378	0.9346	0.9133	0.8909	0.9260	0.9707
Median	0.9510	1	0.9784	0.9508	0.9007	0.9836	1
Maximum	1	1	1	1	1	1	1
Minimum	0.7591	0.8085	0.7666	0.7051	0.7303	0.7792	0.8052
St. Deviation	0.0747	0.0752	0.0854	0.1054	0.1088	0.0862	0.0595
**Frontier hospitals, n (%)**	**6 (46.2%)**	**7 (53.8%)**	**6 (46.2%)**	**6 (46.2%)**	**5 (38.5%)**	**6 (46.2%)**	**9 (69.2%)**

Table S3. Exploratory Second-Stage Simar–Wilson Truncated Regression Results with Double Bootstrap: Model A1

Model A1							
**Variable**	**Coefficient**	**Boot_SE**	**z**	**p_value**	**Sig**	**CI_2.5**	**CI_97.5**
Intercept	2,2023	0,2235	9,854	0	***	1,7532	2,6352
Population ageing	-2,6641	0,5351	-4,979	0	***	-3,709	-1,6138
Population density	-0,0099	0,0018	-5,371	0	***	-0,0136	-0,0062
Unemployment	-3,0686	0,752	-4,081	0	***	-4,4816	-1,5844

Note: *, ** and *** indicate statistical significance at the 10%, 5% and 1% levels, respectively. P-values reported as 0 by the statistical software are presented as p < 0.001

Table S4. Exploratory Second-Stage Simar–Wilson Truncated Regression Results with Double Bootstrap: Model A2

Model A2							
**Variable**	**Coefficient**	**Boot_SE**	**z**	**p_value**	**Sig**	**CI_2.5**	**CI_97.5**
(Intercept)	1,9721	0,2315	8,518	0	***	1,5232	2,427
Population ageing	-2,2811	0,5744	-3,972	0,0001	***	-3,3905	-1,1339
Unemployment	-2,4984	0,8249	-3,029	0,0025	***	-4,1078	-0,8869

Note: *, ** and *** indicate statistical significance at the 10%, 5% and 1% levels, respectively. P-values reported as 0 by the statistical software are presented as p < 0.001

Table S5. Exploratory Second-Stage Simar–Wilson Truncated Regression Results with Double Bootstrap: Model A3

Model A3							
**Variable**	**Coefficient**	**Boot_SE**	**z**	**p_value**	**Sig**	**CI_2.5**	**CI_97.5**
(Intercept)	1,3267	0,2143	6,192	0	***	0,8991	1,7261
Population density	-0.0071	0.0028	-2,501	0,0124	**	−0.0127	−0.0015
Unemployment	-2,9197	1,2103	-2,412	0,0158	**	-5,198	-0,5358

Note: *, ** and *** indicate statistical significance at the 10%, 5% and 1% levels, respectively. P-values reported as 0 by the statistical software are presented as p < 0.001

Table S6. Exploratory Second-Stage Simar–Wilson Truncated Regression Results with Double Bootstrap: Model A4

Model A4							
**Variable**	**Coefficient**	**Boot_SE**	**z**	**p_value**	**Sig**	**CI_2.5**	**CI_97.5**
(Intercept)	1,6731	0,2194	7,627	0	***	1,2471	2,0969
Population ageing	-2,7135	0,691	-3,927	0,0001	***	-4,063	-1,3671
Population density	-0,0079	0,0023	-3,485	0,0005	***	-0,0127	-0,0036

Note: *, ** and *** indicate statistical significance at the 10%, 5% and 1% levels, respectively. P-values reported as 0 by the statistical software are presented as p < 0.001
